# Osmotic and Salt Stresses Modulate Spontaneous and Glutamate-Induced Action Potentials and Distinguish between Growth and Circumnutation in *Helianthus annuus* Seedlings

**DOI:** 10.3389/fpls.2017.01766

**Published:** 2017-10-18

**Authors:** Maria Stolarz, Halina Dziubinska

**Affiliations:** Department of Biophysics, Institute of Biology and Biochemistry, Maria Curie-Skłodowska University, Lublin, Poland

**Keywords:** osmotic potential, salt stress, circumnutation, plant movement, electrophysiology, action potential, electrical transmission, signaling

## Abstract

Action potentials (APs), i.e., long-distance electrical signals, and circumnutations (CN), i.e., endogenous plant organ movements, are shaped by ion fluxes and content in excitable and motor tissues. The appearance of APs and CN as well as growth parameters in seedlings and 3-week old plants of *Helianthus annuus* treated with osmotic and salt stress (0–500 mOsm) were studied. Time-lapse photography and extracellular measurements of electrical potential changes were performed. The hypocotyl length was strongly reduced by the osmotic and salt stress. CN intensity declined due to the osmotic but not salt stress. The period of CN in mild salt stress was similar to the control (~164 min) and increased to more than 200 min in osmotic stress. In sunflower seedlings growing in a hydroponic medium, spontaneous APs (SAPs) propagating basipetally and acropetally with a velocity of 12–20 cm min^−1^ were observed. The number of SAPs increased 2–3 times (7–10 SAPs 24 h^−1^plant^−1^) in the mild salt stress (160 mOsm NaCl and KCl), compared to the control and strong salt stress (3–4 SAPs 24 h^−1^ plant^−1^ in the control and 300 mOsm KCl and NaCl). Glutamate-induced series of APs were inhibited in the strong salt stress-treated seedlings but not at the mild salt stress and osmotic stress. Additionally, in 3-week old plants, the injection of the hypo- or hyperosmotic solution at the base of the sunflower stem evoked series of APs (3–24 APs) transmitted along the stem. It has been shown that osmotic and salt stresses modulate differently hypocotyl growth and CN and have an effect on spontaneous and evoked APs in sunflower seedlings. We suggested that potassium, sodium, and chloride ions at stress concentrations in the nutrient medium modulate sunflower excitability and CN.

## Introduction

Responses to stress and stimuli are essential for adaptation of organisms to environmental conditions. The role of signals propagated along plants organ and organ movements in these responses is an intriguing question. Universal electrical signals, i.e., action potentials (APs) propagating acropetally and basipetally many centimeters along plant organs from cell to cell are well-documented. Complete electrophysiological characterization of APs (threshold, refractory periods, “all-or-none” law, velocity of propagation, chronaxie, reobase) has been carried out for *Helianthus annuus, Lupinus angustifolius*, and *Conocephalum conicum* (Paszewski and Zawadzki, 1973, 1974, 1976a,b; Zawadzki, 1979, 1980; Zawadzki et al., 1991, 1995; Favre et al., 1999). It is known that APs are involved in rapid plant movement and regulate many physiological processes and the circumstances of their appearance are still studied (Sibaoka, 1991; Stankovic et al., 1998; Dziubinska, 2003; Stahlberg et al., 2006; Zimmermann et al., 2009, 2016; Król et al., 2010; Stolarz et al., 2010; Salvador-Recatala et al., 2014; van Bel et al., 2014; Kiep et al., 2015; Macedo et al., 2015; Salvador-Recatala and Tjallingii, 2015; Hedrich et al., 2016; Salvador-Recatala, 2016). A spontaneous action potential (SAP) is an action potential in which exogenous or endogenous stimuli evoking them are not known. Usually, in plants, APs result from an external stimulus (Paszewski and Zawadzki, 1973; Trebacz and Zawadzki, 1985; Favre et al., 1999; Krol et al., 2006; Król et al., 2010). However, some excitable cells do not require stimuli for generation of APs (Zawadzki et al., 1995). They spontaneously depolarize and generate an AP usually at a regular rate/rhythm. This SAP rate/rhythm can be adjusted by environmental condition as was shown recently in *H. annuus* (Stolarz and Dziubinska, 2017). The external stimuli or environmental conditions do not cause SAPs, but merely alter their rate/rhythm. In plants, SAPs were described over 20 years ago by Zawadzki et al. (1995) in *H. annuus* and lately they have been observed in *Solanum lycopersicum* plants (Macedo et al., 2015). The AP ion mechanism in plants has been elaborated in many species, for example *C. conicum, Arabidopsis thaliana*, and *Physcomytrella patens* (Trebacz and Zawadzki, 1985; Trebacz et al., 1989, 1994, 2007; Trebacz, 1992; Krol et al., 2006, 2007; Koselski et al., 2015, 2017). Fundamental players in the AP ion mechanism in plants are H^+^, Ca^2+^, K^+^, and Cl^−^. The latter ones (K^+^ and Cl^−^) are also the major osmotically active elements in the cytoplasm and vacuole and are involved in both osmoregulation and turgor maintenance and thus water fluxes and cell volume changes (White, 2001; Zhu, 2003; Ashley et al., 2006; Shabala and Cuin, 2008; Szczerba et al., 2009; Aleman et al., 2011; Shabala and Pottosin, 2014; Shabala et al., 2015; Coskun et al., 2016). Osmotically driven water fluxes resulting in cell volume changes are fundamental for cell elongation and essential for endogenous movement named circumnutation (CN) (Johnsson, 1979; Millet and Badot, 1996; Shabala and Newman, 1997; Shabala and Knowles, 2002; Shabala, 2003; Stolarz, 2009; Grefen et al., 2011; Kurenda et al., 2015). An endogenous CN can be modulated by multiple external stimuli e.g., light, wounding, touch, temperature, chemicals, and gravity as well as by organ morphology and biological clock (Buda et al., 2003; Hayashi et al., 2004; Charzewska and Zawadzki, 2006; Stolarz, 2009; Stolarz et al., 2010). CNs are an effect of highly coordinated and phase synchronized cell elongation and intercellular communication inside the motor/elongation zone of a growing organ. The membrane potential changes and ion fluxes are important elements of the CN mechanism (Millet and Badot, 1996; Stolarz, 2009; Kurenda et al., 2015). The ion content in the soil (nutrient solution), ion uptake, and ion content in the apoplast and symplast is therefore essential for transmembrane potential maintenance; it could change plant excitability and cell volume and growth and thus CN. Growth inhibition in plants is a most frequently defined effect of environmental stress, including salt stress (Zhu, 2003; Foster and Miklavcic, 2015; Wu et al., 2015a). CNs are closely related to growth, and CN changes after many environmental stimuli have been described (Buda et al., 2003; Stolarz et al., 2008, 2015; Stolarz, 2009; Kurenda et al., 2015). An endogenous motor activity and electrical long-distance signaling in the form of CNs and APs have been well-characterized in *H. annuus*. *H. annuus* is an important agricultural crop grown for oil, fresh green mass, and as ornamental plants whose yield can be reduced by drought and the accompanying osmotic and salt stress. The sunflower is studied simultaneously as a crop with relatively high tolerance to drought and therefore osmotic and saline stress (Mukherjee et al., 2014; Ceccoli et al., 2015; Wu et al., 2015b; Singh and Bhatla, 2016). Investigations of the effect of the osmotic potential of the nutrient medium on the transmission of electrical signals and changes in endogenous motor activity of the sunflower may provide new information on the role of intercellular communication in plant adaptation to changing environmental conditions.

The aim of our study was to characterize the effect of osmotic and salt stress on growth, CN parameters, and appearance of APs. A different effect of salt stress than osmotic stress on CN and, hence, on the growth and appearance of APs was revealed. For the first time, spontaneous APs in seedlings growing in a hydroponic medium and enhancement of spontaneous excitation in sunflower seedlings by salinity were shown.

## Materials and methods

### Experimental plants

#### Sunflower seedlings

Seeds of *H. annuus* L. (PNOS, Ozarów Maz., Poland) were germinated on wet filter paper in a thermostatted (24 ± 1°C) darkened chamber. After 4 days, seedlings with 4.5 ± 0.5-cm-long hypocotyls were cultivated hydroponically (10 plants per pot) in an aerated nutrient solution. The hydroponic culture was maintained for 3 days under constant illumination, 40 μmol m^−2^s^−1^ white light (Power Star HQT-T400 W/D OSRAM GmbH, Munich, Germany), at a temperature of 24 ± 1°C and relative humidity 50–70%. The seedlings were grown for 3 days in a control medium or were treated with different KCl, NaCl, and D-sorbitol concentrations and simultaneously filmed for CN measurements. In 7-day-old seedlings, the length of the seedling hypocotyl was measured and electrophysiological measurements of the seedlings were performed.

#### Three week-old sunflowers

The studies were carried out on 3 week-old *H. annuus* L. plants (PNOS, Ożarów Maz. Poland) grown in a vegetation room in pots filled with garden soil. They were watered with tap water and no other treatment was applied. A 16:8 h light:dark (4:00 a.m.–8:00 p.m.) photoperiod was maintained. The intensity of white light in the PAR (Photosynthetic Active Radiation) range (Power Star HQT-T400 W/DOSRAM GmbH, Munich, Germany) at the level of plant leaves was ~70 μmol m^−2^s^−1^. The vegetation room was air-conditioned; the temperature was 24 ± 1°C and humidity 50–70%. Approximately 20–30 cm high plants with one or two pairs of developed leaves were taken for the experiments. The 3-week old plants were transferred to a Faraday cage at ~12:00 and electrodes were inserted. Distilled water, D-sorbitol, KCl, or NaCl were injected between 6:00 p.m.–7:00 p.m.

### Time-lapse method and CN measurements

For CN measurements, time-lapse photography recordings were made from 09:00 a.m. on the fourth day to 09:00 a.m. on the seventh day of seedling growth. A monochromatic camera (Mintron MTV-1368CD, Mintron Enterprise Co. Ltd, Taipei, Taiwan) was used to record the circumnutation trajectory of the hypocotyls apex. The plants were filmed from the top. Time-lapse images were recorded one frame per 5 min by Gotcha! Multicam software (Prescient System Inc., West Chester, PA, USA). The system was calibrated in a millimeter scale. The time-lapse images were digitized using *Circumnutation Tracker* (Stolarz et al., 2014) and Microsoft Excel programs. Experimental points (coordinates *x, y* of the stem apex on the horizontal plane) were determined at 5-min intervals. In the geographic direction plane, the single circumnutation cycle is determined by two subsequent maximum northward bends of the organ (Stolarz et al., 2014). Supplementary Video S1 shows subsequent CN cycles of sunflower seedlings. The distance covered by the hypocotyls apex during one CN cycle was used to calculate the CN rate. CN intensity was the rate of CN divided by the length of the hypocotyls. The CN period was the time required for the hypocotyls apex to trace a single CN cycle (time between two subsequent maximum northward bends of the organ) (Stolarz et al., 2014).

### Electrophysiological measurements—extracellular method

The electrical measurements were carried out in a Faraday cage on 7- to 8-day-old seedlings or 3-week-old sunflowers. The changes in the electrical potential were measured with two or four extracellular Ag/AgCl electrodes (a silver wire, 0.2 mm diameter, World Precision Instruments, Sarasota, FL, USA) inserted across the sunflower hypocotyl or stem and then interfaced with a multi-channel data acquisition system composed of a differential amplifier (ME-4600 Meilhaus, Germany) and RealView software (Abacom, Germany) (Supplementary Figure S1). During the preparation of the Ag/AgCl electrodes, the silver wire was electrolytically coated with silver chloride. The electrical potential was recorded from tissues adjacent to the electrode, i.e., vascular bundles, parenchyma, and epidermis (Dziubinska et al., 2001). The reference electrode (Ag/AgCl) was placed in the hydroponic medium or soil. The frequency of sample recording was 1 Hz. For registration of SAPs (spontaneous action potentials), seedlings grew in the Faraday cage in a hydroponic solution for 3 days. Glutamate (Glu) injection was applied between 8:00 a.m.–2:00 p.m. in 7–8-day-old seedlings growing in different nutrient solutions for 3 days.

### Chemicals

#### Hydroponic culture

The nutrient solution contained 4 mM Ca(NO_3_)_2_ × 4H_2_O, 5 mM KNO_3_, 1 mM NH_4_H_2_PO_4_, 2 mM MgSO_4_ × 7H_2_O; microelements: 0.085 mM Fe(III)citrate, 0.046 mM H_3_BO_3_, 0.0009 mM MnCl_2_ × 4H_2_O, 0.0003 mM CuSO_4_ × 5H_2_O, 0.0008 mM ZnSO_4_ × 7H_2_O, 0.0001 mM H_2_MoO_4_ × 2H_2_O; (pH 6.0); the osmotic potential was 23 mOsm. Additionally, 80 mM (160 mOsm), 120 mM (240 mOsm), and 160 mM (300 mOsm) potassium chloride (POCH, Poland) and sodium chloride (POCH, Poland) in the nutrient solution was used as a hyperosmotic salt stress. Hyperosmotic stress was adjusted by 160 mM (160 mOsm) D-sorbitol (POCH, Poland). The osmotic potential was measured with a cryoscopic osmometer (Osmomat 030, Gonotec GmbH, Berlin, Germany).

#### Injection

Twenty microliters of a 50 mM Glu solution (l-glutamic acid, ICN Biomedicals, Germany, pH adjusted to 7 by Tris/Mes buffer), distilled water, 250 mM KCl (500 mOsm) (POCH, Poland), 250 mM NaCl (500 mOsm) (POCH, Poland), and 500 mM D-sorbitol (500 mOsm) (POCH, Poland) were injected with a syringe into the seedlings at the base of the hypocotyls (Glu), 1 cm above the root collar, or at the base of the stem in the 3-week old plants. The injection of the solution lasted a few seconds. The injury of the sunflower hypocotyls or stem with a syringe needle induced either only one AP or none (Stolarz et al., 2010).

### Statistical analysis

The data were analyzed using Statistica ver. 12 software (StatSoft, Inc., 2014). The data set was first tested for normality using Shapiro-Wilk test and homogeneity of variance by Levene's test. The non-parametric Kruskal-Wallis ANOVA and non-parametric Mann-Whitney *U*-test for pairwise analysis were used when the data had non-normal distribution or unequal variance. The one-way ANOVA and *post-hoc* Tukey test for pairwise analysis were used when the data had normal distribution and equal variance. The level of statistical significance for all tests was set at *p* < 0.05.

## Results

The sunflower seedlings treated with hypoosmotic (distilled water) and hyperosmotic (D-sorbitol, KCl and NaCl) nutrient solutions in the hydroponic culture were characterized by decreased growth and changes in the CN parameters. Spontaneous excitation was also modified by the osmotic potential of the nutrient solution. Additionally, localized stimuli—the injection of the glutamate solution at the base of hypocotyls or the hypo- or hyperosmotic solution at the base of the stem induced different numbers of APs dependent on the kind of the injected solution and the osmotic potential of the nutrient.

### Effect of osmotic and salt stress on the growth and CN intensity in sunflower seedlings

**Figure 1:**
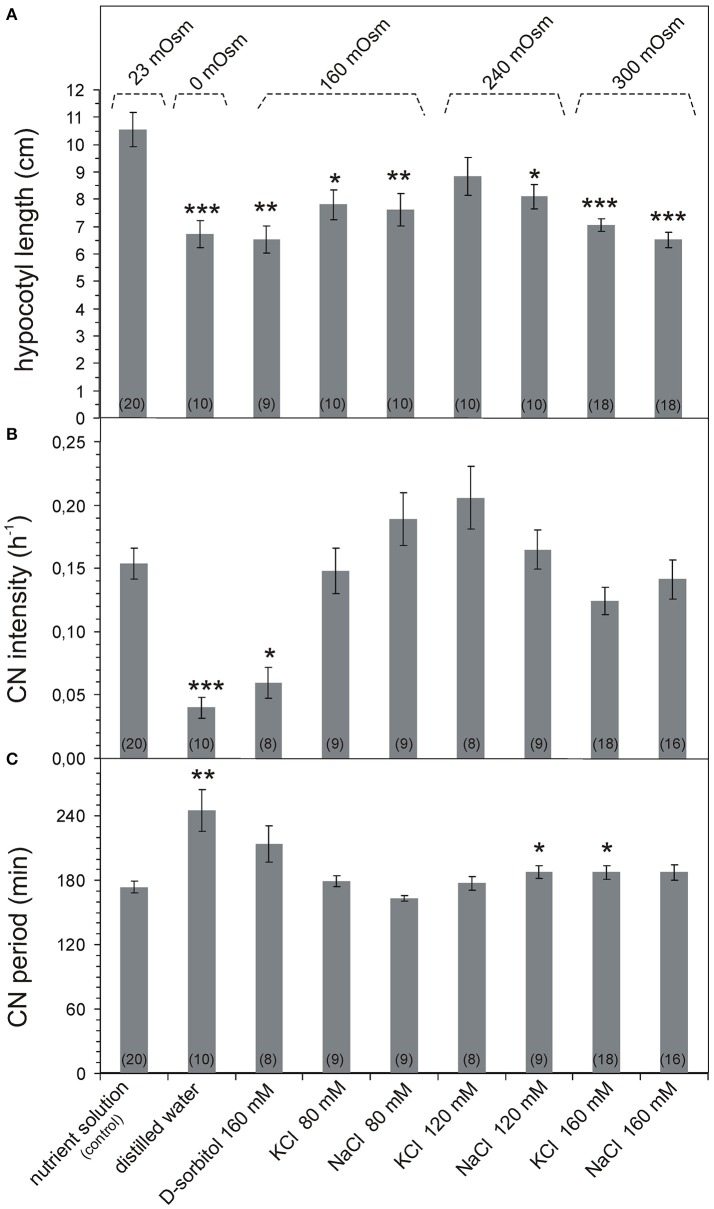
Parameters of growth and CN of *Helianthus annuus* seedlings under osmotic and salt stresses. Bars represent mean ± SE. The number of plants is indicated in parentheses. The data were tested for normality distribution using the Shapiro-Wilk test. The the non-parametric Mann-Whitney *U*-test for pairwise analysis **(A,C)** and Tukey test **(B)** were used to assess the statistical difference between the control plants and each sunflower group growing in different nutrients; *p*-value ranges are marked by asterisks: ^***^p < 0.001,^**^p < 0.01,^*^p < 0.05. **(A)** Changes in hypocotyl length, data normally distributed, unequal variance (Levene's test *p* = 0.000109), Kruskal-Wallis ANOVA (Chi square = 27.39 df = 8 *p* = 0.0006). **(B)** Changes in hypocotyl CN intensity. The distance covered by the hypocotyl apex during one cycle was used to calculate the CN rate. CN intensity was the rate of CN divided by hypocotyl length. Data normally distributed, equal variance Levene's test (*p* = 0.30), one-way ANOVA (SS = 0.2149 df = 8 F = 10.015 *p* = 0.0000). **(C)** Changes in hypocotyl CN period. The CN period was the time required by the hypocotyl apex to trace a single CN cycle (time between two subsequent maximum northward bends of the hypocotyl). Data normally distributed, unequal variance (Levene's test *p* = 0.000053), Kruskal-Wallis ANOVA (Chi square = 24.55 df = 8 *p* = 0.0018).

The sunflower seedlings growing in the control nutrient solution (23 mOsm) had 10 ± 0.6 cm (*n* = 20) long hypocotyls. Both the hypoosmotic and hyperosmotic nutrient solutions reduced the hypocotyl length in a statistically significant way by ~10–30% (Figure 1A, Supplementary Figure S2). Simultaneously, the CN parameters changed differently in the osmotic and salt stress conditions. Sunflower seedlings growing in distilled water (0 mOsm) had significantly shortened hypocotyls and drastically reduced CN intensity in relation to those growing in the control nutrient solution (Figures 1A,B). Similarly, the high osmotic potential (160 mOsm) evoked by D-sorbitol significantly shortened the hypocotyl length and drastically reduced CN intensity (Figures 1A,B). In turn, the increase in the osmotic potential to 160–300 mOsm induced by salt (KCl or NaCl) did not influence the CN intensity although the hypocotyl length was significantly reduced (Figures 1A,B). Sunflower seedlings with similar hypocotyl length exhibited completely different CN intensity in osmotic and salt stress. The CNs were strongly inhibited in distilled water and 160 mM D-sorbitol (160 mOsm) but were vigorous (as in the control seedlings) in the salt stress inducing solutions (160–300 mOsm KCl and NaCl). The osmotically induced growth inhibition was accompanied by a decrease in the CN intensity but the salt-induced growth inhibition had little effect on the CN intensity.

### Effect of osmotic and salt stress on the CN period in sunflower seedlings

In the sunflower seedlings, the mean CN period in the control solution was 174 ± 6 min (*n* = 20, Figure 1C). The CN period was lengthened in a statistically significant way in seedlings growing in distilled water (0 mOsm) to 245 ± 19 min (*n* = 10). The CN period was longer by 70 min. Simultaneously, the CN period in seedlings growing in the D-sorbitol, KCl, and NaCl solutions (160–300 mOsm) was the same as in the control conditions. In 120 mM NaCl and 160 mM KCl, it was significantly longer but only by ~15 min. The CN period was the same as in the control in the mild salt stress and even in the strong stress (160 mM NaCl). The hypocotyls of the same length had a completely different CN period in the osmotic and salt solution, which proves the absence of a strict connection between the CN and hypocotyl growth.

Given the above results, it can be assumed that only the osmotic stress disturbs growth and CN (intensity and period); in turn, the salt stress disturbs the growth mechanism only and does not influence the CN mechanism. This shows that there is a salt stress-resistant ultradian pacemaker in the sunflower and confirms that the presence of ions in the nutrient solution is basic for the CN mechanism.

K^+^, Cl^−^, and Na^+^ are also essential for membrane potential maintenance and excitability of organisms; therefore, we expect that the sunflower seedlings growing under different K^+^, Cl^−^, and Na^+^ contents in the nutrient solution could have a different excitability.

### Spontaneous action potentials in sunflower seedlings

**Figure 2:**
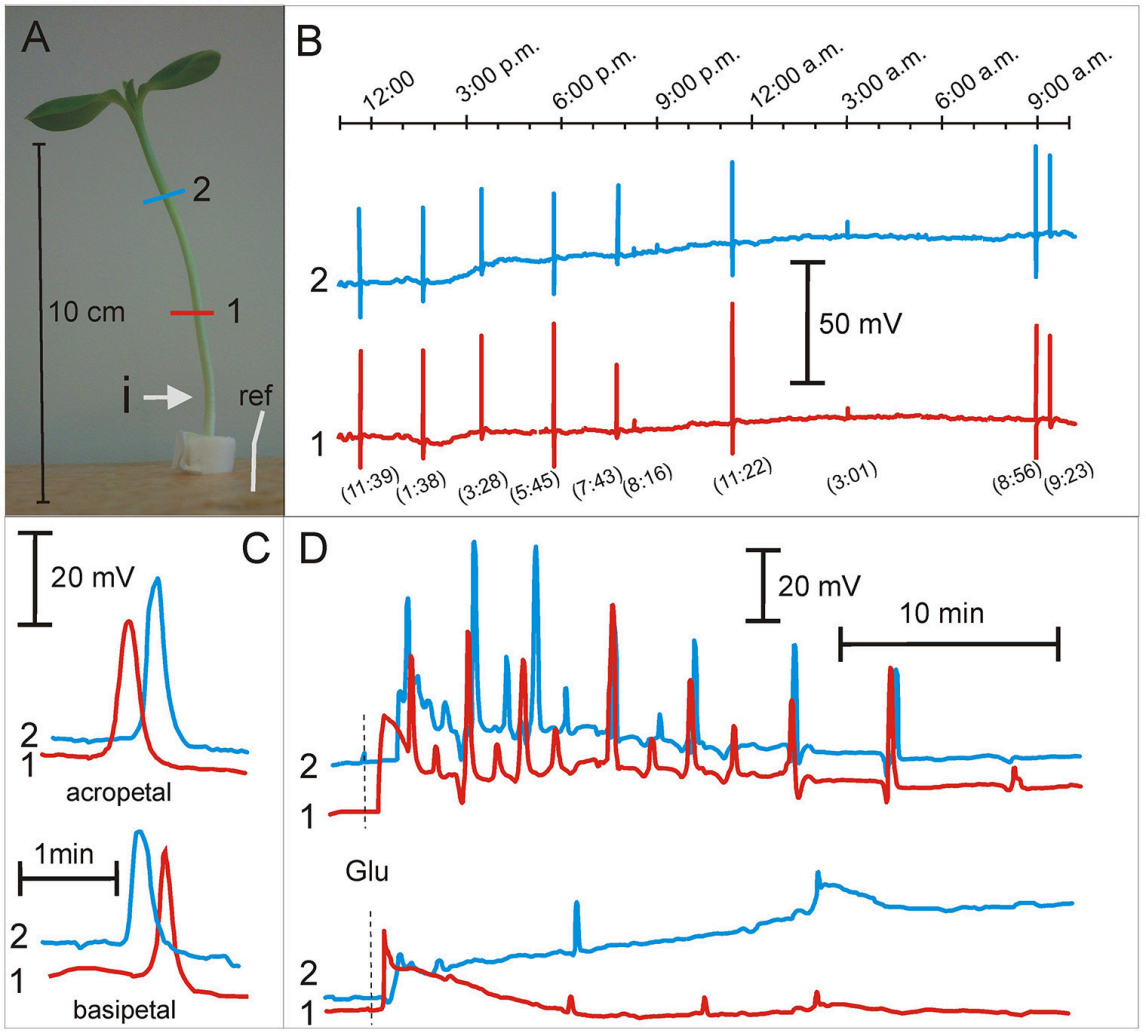
Spontaneous action potentials and glutamate induced series of action potentials in *Helianthus annuus* seedlings under osmotic and salt stresses. **(A)** *Helianthus annuus* seedlings, electrode arrangement (1, 2, ref—reference electrode) and site of glutamate injection (i). **(B)** Example of recordings of spontaneous action potentials during 1 day (also shown in Supplementary Video S2). In the parentheses, the time of SAP appearing. **(C)** Example of recordings of acropetally and basipetally propagating spontaneous action potentials. **(D)** Example of recordings of action potential series after glutamate (Glu) injection into the hypocotyl base of seedlings growing under 80 mM KCl (upper) as well as 160 mM NaCl nutrient solutions (lower). Data details are presented in Tables 1, 2.

The number, velocity, and direction of propagation of SAPs were determined in salt and osmotically stressed sunflower seedlings (Figure 2, Table 1). An example of SAPs in sunflower seedlings is shown in Figure 2 and Supplementary Video S2. In the control conditions, 3 ± 1 SAPs 24 h^−1^ plant^−1^ (*n* = 12) were registered, which propagated mainly basipetally (75%) with a mean velocity 14 ± 1 cm min^−1^. This was similar in the strong salt stress conditions (300 mOsm KCl and NaCl) (Table 1). In the seedlings growing in distilled water and in D-sorbitol (160 mOsm), SAPs were completely silenced. The number of SAPs significantly increased in mild KCl stress where the number of SAPs increased two and three times to 10 SAPs 24 h^−1^ plant^−1^, compared to the control and 300 mOsm salt solution. Similarly, in mild NaCl stress, the SAP number slightly increased to 7 SAPs 24 h^−1^ plant^−1^ and SAP propagation velocity significantly increased to 20 ± 1 cm min^−1^ in those seedlings. The percentage of basipetally and acropetally propagating SAPs remained similar in all conditions (Table 1). The complete lack of SAPs in distilled water and the 160 mOsm D-sorbitol solution as well as the significantly increased number of SAPs and their propagation velocity in mild salt stress showed that the K^+^, Cl^−^, and Na^+^ concentration in the nutrient solution modulated spontaneous excitability. The silencing of SAPs and the reduced vigor of CNs indicates that SAPs and CNs were repressed by the lack of ions or ion uptake disturbance in distilled water and the hyperosmotic D-sorbitol solution. Mild and strong salt stress nutrient conditions maintain CNs and maintain or even enhance SAPs; thus, these results show an essential role of K^+^, Cl^−^, and Na^+^ ions in both phenomena.

**Table 1:** Parameters of spontaneous excitation in *Helianthus annuus* seedlings under osmotic and salt stresses.

**Hydroponic medium (mM)**	**Number of studied plants**	**% of excitable plants**	***n***	**SAPs 24 h^−1^ plant^−1^**	**SAPs velocity (cm min^−1^)**	**SAPs direction of propagation %**	
						**basipetal**	**acropetal**
Distilled water	8	0	0	lack of SAPs			
Nutrient solution (control)	12	75	9	3 ± 1	14 ± 1	75	25
160 mM D-sorbitol	6	0	0	lack of SAPs			
80 mM KCl	12	100	12	10 ± 2^***^	14 ± 1	77	23
80 mM NaCl	8	62	5	7 ± 2	20 ± 1^***^	71	29
160 mM KCl	8	100	8	4 ± 1	15 ± 1^*^	75	25
160 mM NaCl	8	75	6	4 ± 1	12 ± 1	80	20

*Propagation velocity of APs was calculated by taking the distance between the electrodes and dividing it by the time necessary for AP to move between the electrodes. Basipetal SAPs are SAPs propagating downwards the stem; acropetal SAPs are SAPs propagating upwards the stem; n—number of seedlings with SAPs*.

SAPs 24 h^−1^ plant^−1^: data non-normally distributed, unequal variance (Levene's test p = 0.000022), Kruskal-Wallis ANOVA (Chi square = 10.31 df = 4 p = 0.0354). SAP velocity: data non-normally distributed, unequal variance (Levene's test p = 0.000022), Kruskal-Wallis ANOVA (Chi square = 33.66 df = 4 p = 0.0000). Mann-Whitney U-test was used to assess the statistical difference between the control plants and each sunflower group growing in different nutrients. p-value ranges are marked by asterisks:

*** *p < 0.001*,

* *p < 0.05*.

### Glu-induced series of APs in the hypocotyls of sunflower seedlings

Besides SAPs, Glu-induced series of APs in osmotically and salt-stressed seedlings were studied. The series of APs evoked by injection of the 50 mM Glu solution in the control plants consisted of 10 ± 1 APs (*n* = 10) (Figure 2D, Table 2). Similar series were obtained in mild stress in the D-sorbitol, KCl and NaCl (160 mOsm) treated seedlings; however, in strong KCl and NaCl (320 mOsm) stress, the series were significantly inhibited to 2–3 APs in a series (Figure 2D, Table 2). Seedlings growing in distilled water exhibited reduced growth, CNs (Figures 1A,B), and Glu-induced series of APs (only 30% of the seedlings were excitable, Table 2), compared to the control plants. Seedlings growing in D-sorbitol (160 mOsm) had shorter hypocotyls and reduced CN but generated Glu-induced series, likewise the control and mild stress-treated plants, which had shorter hypocotyls and unchanged vigor of CN. Additionally, sunflower seedlings treated with strong salt stress (300 mOsm) had a reduced hypocotyl length and unchanged CN, but the Glu-induced series of APs were strongly inhibited.

**Table 2:** Parameters of glutamate induced series of action potentials in *Helianthus annuus* seedlings growing under osmotic and salt stresses.

**Hydroponic medium (mM)**	**Number of studied plants**	**% of excitable plants**	***n***	**APs series**	
				**APs number**	**Series duration (min)**
Distilled water	7	29	2	5 ± 2	13 ± 2
Nutrient solution (control)	10	100	10	10 ± 1	20 ± 5
160 mM D-sorbitol	14	100	14	10 ± 1	22 ± 5
80 mM KCl	16	100	16	11 ± 1	35 ± 3^*^
80 mM NaCl	8	50	4	7 ± 1	16 ± 5
160 mM KCl	12	58	7	2.4 ± 0.2^***^	9 ± 2
160 mM NaCl	13	69	9	2.8 ± 0.3^***^	33 ± 15

The duration of the series is the time between the first and the last AP in the series. n—number of excitable plants. AP number: data non-normally distributed, unequal variance (Levene's test p = 0.0020), Kruskal-Wallis ANOVA (Chi square = 27.22 df = 6 p = 0.0001). Series duration: data non-normally distributed, unequal variance (Levene's test p = 0.0421), Kruskal-Wallis ANOVA (Chi square = 13.57 df = 6 p = 0.0421). Mann-Whitney U-test was used to assess the statistical difference between the control plants and each sunflower group growing in different nutrients. p-value ranges are marked by asterisks:

*** *p < 0.001*,

* *p < 0.05*.

The spontaneous and glutamate-induced excitability changes in seedlings growing in the osmotic and salt stress conditions encouraged us to check the APs appearing after injection of the hypoosmotic and hyperosmotic solution in 3-week old sunflower plants.

### Osmotically and salt-induced series of APs in the stem of 3-week old sunflowers

**Figure 3:**
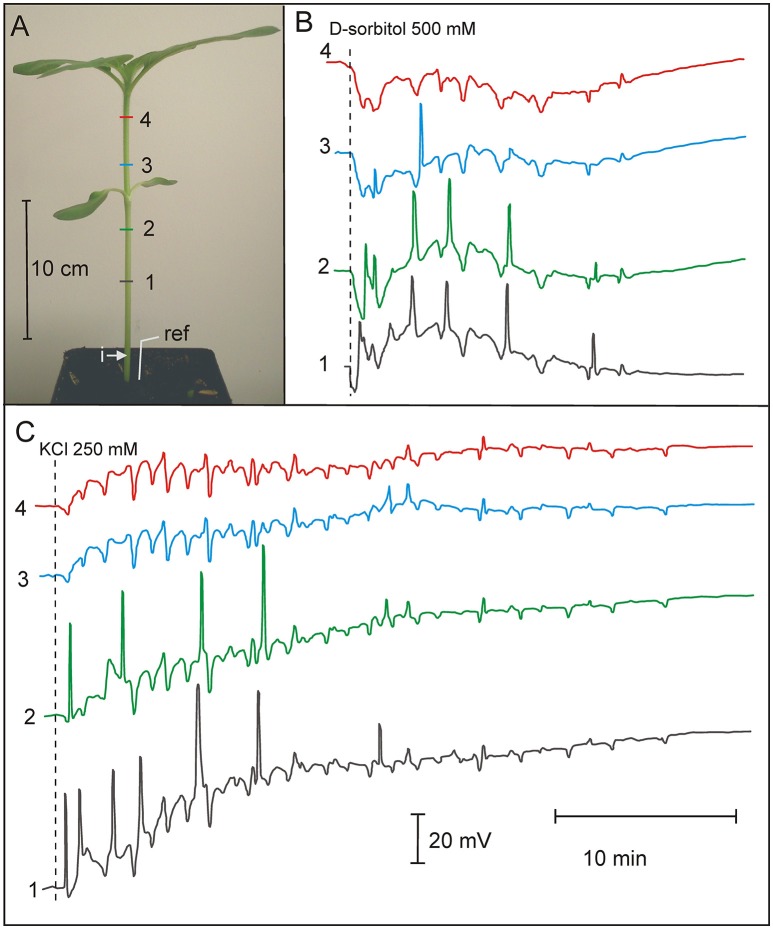
Osmotically and potassium chloride-induced series of action potentials in 3-week old *Helianthus annuus*. **(A)** *Helianthus annuus* plants, electrode arrangement (1, 2, 3, 4, ref—reference electrode) and site of solution injection (i). **(B)** Example of recordings of action potential series after D-sorbitol 500 mOsm (500 mM) injection into the stem base. **(C)** Example of recordings of action potential series after KCl 500 mOsm (250 mM) injection into the stem base. Data details are presented in Table 3.

Injection of distilled water or 500 mOsm solutions (D-sorbitol, KCl) into the sunflower stem evoked series of APs presented in Figure 3. The parameters of sunflower excitation after injection of the osmotic and salt solutions are presented in Table 3. Distilled water, D-sorbitol, and KCl solutions evoked series of 3 to 24 APs in 70–100% of treated plants. APs propagated mainly acropetally from the site of injection toward the stem apex, at an ~15 cm long distance. The injection of the NaCl 250 mM solution did not evoke APs (Supplementary Figure S3). These results show that a series of long-distance propagating (~15 cm) APs in the sunflower stem can be induced by distilled water, D-sorbitol and KCl but not by NaCl.

**Table 3:** Parameters of osmotic and salt-induced series of action potentials in 3-week old *Helianthus annuus*.

**Solution injected**	**Number of treated plants**	**% of excitable plants**	***n***	**Series of AP**	
				**APs number**	**Series duration (min)**
Distilled water 0 mOsm	9	70	6	6 ± 1^a^	11 ± 2^a^
D-sorbitol 500 mM 500 mOsm	8	100	8	8 ± 1^a^	13 ± 1^a^
KCl 250 mM 500 mOsm	7	100	7	17 ± 1^b^	27 ± 2^b^
NaCl 250 mM 500 mOsm	10	0	0	lack of series of APs	

*The duration of the series is the time between the first and the last AP in the series. n—number of excitable plants. AP number: data normally distributed, unequal variance (Levene's test p = 0.0025), Kruskal-Wallis ANOVA (Chi square = 13.48 df = 2 p = 0.0012). Series duration: data normally distributed, unequal variance (Levene's test p = 0.0017), Kruskal-Wallis ANOVA (Chi square = 17.29 df = 2 p = 0.0002). Mann-Whitney U-test was used to assess the statistical differences in one parameter between the groups; different letters denote statistical difference*.

## Discussion

Closure of the trap leaf in *Dionaea* and leaf folding in *Mimosa* after touching are commonly known, easily observable examples of plant responses to environmental stimuli, in which the universal biological signal AP is involved. Thanks to the recent well-developed time-lapse photography method, slow endogenous organ motion can be visualized and investigated under stimuli and environmental changes (Buda et al., 2003; Stolarz et al., 2003, 2014; Kurenda et al., 2015). Long lasting extracellular electrical measurements can show also plant excitability in a wide time span of even a few days (Zawadzki et al., 1995; Favre et al., 1999; Macedo et al., 2015). For better understanding of the role of APs as physiological signals in plants, SAPs and Glu-induced excitation was studied in osmotically and salt-stressed sunflower seedlings in relation to hypocotyl growth and CN.

### Hypocotyl elongation, but not CN intensity, was decreased by salinity stress

The problem of CN dependence on growth has been considered many times (Spurny, 1975; Johnsson, 1979, 1997; Millet and Badot, 1996; Stolarz et al., 2008). In general, CNs are dependent on growth, but in some environmental conditions, for example under lithium, red light, or ethylene treatment, growth and CN uncoupling was observed (Spurny, 1968; Zachariassen and Johnsson, 1988; Millet and Badot, 1996; Yoshihara and Iino, 2005; Binder et al., 2006; Stolarz et al., 2008, 2015). This study has shown that the osmotic and salt stress treatments distinguish between growth and CN behavior (Figures 1A,B). Significant inhibition of elongation of hypocotyls was observed when distilled water, D-sorbitol, KCl, and NaCl were applied (Figure 1A). The CN intensity decreased significantly only in seedlings growing in distilled water and D-sorbitol (Figure 1B). Seedlings with a similar hypocotyl length had different CN intensity in osmotic and salt stress. These results showed that the mechanism of elongation is influenced differently than the mechanism of CN. The concentration of 80–160 mM K^+^, Na^+^, and Cl^−^ decreased growth but did not affect the CN intensity. The absence of ions or ion uptake disturbance in distilled water or D-sorbitol inhibited growth and CN.

Some other studies showed the different physiological effect of the “osmotic” and “ionic” component of salt stress. The “osmotic” and “ionic” component of salt stress differently modulated net ion fluxes in leaf mesophyll of *Vicia faba* (Shabala, 2000). Osmotic stress in barley regulated expression of a different set of genes than salt stress (Ueda et al., 2004). The effect of osmotic stress was also shown in *Beta vulgaris* when D-sorbitol-induced osmotic stress strongly reduced its growth and led to a significant decrease in shoot osmotic potential, water content, and K^+^ concentrations (Wu et al., 2015a).

### Stable CN period in salt stress

Circumnutation, a rhythmic phenomenon can be considered as a “cue” internal ultradian oscillator (Millet and Badot, 1996; Shabala, 2003; Lloyd, 2006). The CN period and it shortening or lengthening in various environmental conditions is important in a study of the ultradian pacemaker mechanism (Johnsson, 1979, 1997; Zachariassen and Johnsson, 1988; Stolarz, 2009; Hinrichsen et al., 2012). In this study, a stable period, despite the growth reduction, was shown (Figures 1A,C). The period of CNs in the mild and strong salt stress (163–188 min) was close to the CN period of the control plants (174 min). The CN period was lengthened significantly only in distilled water (245 min). This confirms a strong resistance of the CN period to osmotic and salt stress (Erdei et al., 1998). The independence of the CN period of the growth of hypocotyls confirmed some degree of independence between growth and CN behavior, as in the case of lithium treatment (Stolarz et al., 2015).

The results presented above confirmed again the autonomy of CN behavior in relation to elongation and special physiological and ecological functions of CN being not merely a strict result of growth (Darwin and Darwin, 1880; Inoue et al., 1999; Larson, 2000; Kosuge et al., 2013; Migliaccio et al., 2013).

### Mild salt stress enhances spontaneous action potentials in sunflower seedlings

In plants, beside the stimulus-induced APs (electrical and mechanical stimuli, light, temperature, chemicals), there are SAPs. The SAPs appear in the absence of external stimuli and their endogenous source is unknown. SAPs were previously observed in 3-week old *Helianthus* and *Lycopersicum* plants growing in pots with standard soil (Zawadzki et al., 1995; Macedo et al., 2015). In this study, we have shown for the first time SAPs in sunflower seedlings growing in a hydroponic medium (Figure 2, Supplementary Video S2). This offered a new opportunity to modify the nutrient composition and investigate SAPs in plants in a specified nutrient environment.

In this work, we have described the number of SAPs per 24 h^−1^plant^−1^ as a parameter of endogenous “firing,” which we have shown to be able to appear in control conditions (no external stimuli) but were modulated by osmotic and ionic stress. In the controlled nutrient conditions, the sunflower seedlings generated 3 ± 1 SAPs 24 h^−1^plant^−1^ (*n* = 12). This number of SAPs persisted even under strong salt stress but completely disappeared in the distilled water and D-sorbitol (160 mM, 160 mOsm) treatment (Table 1). The number of SAPs increased significantly to 10 ± 2 SAPs 24 h^−1^ plant^−1^ (*n* = 12) in 80 mM KCl (160 mOsm). This showed that the “ionic” but not “osmotic” component of salt stress increased the number of SAPs. The number of SAPs 24 h^−1^ plant^−1^ differed in the different nutrient conditions. These results demonstrate that sunflower seedlings are capable of generating many APs, and in a sufficiently long time, we can consider this as a specific frequency code of APs. In slowly moving hypocotyls of sunflower seedlings, this approach is justified. Slow motion of hypocotyls is accompanied by low frequency electrical signals (from 1 to 45 of APs during 24 h per seedling). In such a long time span, there are various numbers of SAPs and this amount can be modulated by the nutrient environment; therefore, there is endogenous electrical activity with very low frequency coding in sunflower seedlings. SAPs are known to propagate acropetally and basipetally. Basipetally propagating SAPs dominated in all nutrient solutions. They appeared in the upper part of the seedlings and propagated downwardly. This showed that the upper part of the seedlings generated more SAPs than the lower part. The amplitudes were in the range of 5–60 mV and the rate of propagation was 12–20 cm min^−1^. In evoked APs, the amplitude is relatively constant (50–60 mV). Significantly lower amplitudes of some SAPs indicate that the excitation is transmitted along individual phloem vessels but not along the transmitting system as in the case of evoked APs. Seedlings growing in the mild NaCl stress exhibited significantly higher SAP propagation velocity than the control plants. The amplitude and velocity of propagation was similar to that in 3-week old *Helianthus* and *Lycopersicum* plants growing in pots with standard soil (Zawadzki et al., 1995; Macedo et al., 2015).

In the research of changes in excitability in sunflowers, we used KCl and NaCl. A treatment with the KCl solution was applied because K^+^ is an important component of the AP mechanism and transmembrane potential maintenance (Trebacz et al., 1989, 1994; Shabala, 2003; Johansson et al., 2006; Shabala and Cuin, 2008; Aleman et al., 2011; Dreyer and Uozumi, 2011; Shabala and Pottosin, 2014; Salvador-Recatala, 2016). In turn, NaCl was used because it is the most commonly occurring salt in the environmental salt stress (Munns and Tester, 2008; Maathuis, 2014; Foster and Miklavcic, 2015). The mechanism of the action of environmental salt (NaCl) stress on plants also includes K^+^ homeostasis disturbance (Shabala and Cuin, 2008; Foster and Miklavcic, 2015). The effect of KCl and NaCl on SAP appearance was different (Table 1). The mild stress induced by KCl raises the number of SAPs 24 h^−1^ plant^−1^ in a statistically significant way, while NaCl slightly increases this value. The strong stress caused by NaCl and KCl keeps SAPs at a control level. This shows that only the mild stress induced by KCl increases the appearance of SAPs in sunflower seedlings. Potassium ions are an important part of the mechanism of maintaining the excitability of the cell membrane and we can assume that its slight increase in the environment affects the membrane transport (Trebacz et al., 1989, 1994; Shabala, 2003; Johansson et al., 2006; Shabala and Cuin, 2008; Shabala and Pottosin, 2014; Salvador-Recatala, 2016). The mild stress induced by NaCl slightly increases the number of SAPs and increases SAP velocity in a statistically significant way. An increase in SAP velocity occurs also in strong stress induced by KCl. It is presumed that both these effects are due to the involvement of K^+^ and Na^+^ ions in membrane transport and the function of cation channels. The increased velocity of SAP propagation is in accordance with the cable theory, which postulates such an effect after lowering of the external (apoplast) electrical resistance caused by the applied ions.

Additionally, we have shown changes in the growth and intensity of the endogenous movement of the hypocotyl in spontaneously excited seedlings. The complete lack of SAPs in distilled water and D-sorbitol (with an ability to generate Glu-induced APs) growing seedlings with strongly inhibited CN may be associated with CN inhibition. In our recent work, the relationship between SAPs and CN was shown, i.e., the number of SAPs decreased with the restricted CN (Stolarz and Dziubinska, 2017). On the other hand, the similarly circumnutating seedlings had a significantly lower number of SAPs in the strong salt stress [4 ± 1 SAPs 24 h^−1^ plant^−1^ (*n* = 8)] than in the mild salt stress [10 ± 2 SAPs 24 h^−1^ plant^−1^ (*n* = 12)]. This confirms a possible complex relation between endogenous APs and endogenous hypocotyl movements in sunflower seedlings.

### Osmotically and salt-induced series of action potential in 3-week old sunflower

A treatment with KCl combined with prior pricking induced series of APs in *C. conicum* (Favre et al., 1999) and *A. thaliana* (Favre et al., 2011). Recently, Salvador-Recatala (2016) used NaCl and KCl solutions to evoke depolarization of root cells of *A. thaliana*. The salts were also administered in *Vicia faba* and *Hordeum vulgare* to evoke system potentials (SPs) (Zimmermann et al., 2009). An electrical response after the hypo- or hyperosmotic solution injection (Figure 3, Table 3, Supplementary Figure S3) was dependent on the kind of treatment. Usually, a series of APs appeared, but we assume that some of the recorded spikes (Figure 3, electrode 3 and 4) are SPs. A similar recording was shown in *Vicia* faba, *Hordeum vulgare*, and *Nicotiana tabacum* after wounding as well as abiotic and biotic stress (Zimmermann et al., 2009, 2016). The injection of NaCl did not evoke series of APs (Supplementary Figure S3). It is probable that the presence of K^+^ (but not Na^+^) decreases the membrane potential difference and the excitation threshold, which facilitates the generation of APs.

In this study, osmotically driven APs propagating for many centimeters along the sunflower stem have been shown for the first time. Moreover, we have shown that not only salt stimuli but also distilled water and osmotically active solutions (D-sorbitol) induce electrical signals propagating along stem.

Our working hypothesis is that osmotically active solutions and salt solutions affect membrane transport and thus evoke propagated electrical signals and changes in CN. APs could result from adjustment of membrane polarization of some cells over the threshold by ions (K^+^, Na^+^, and Cl^−^) contained in the solutions (in the nutrient or injected). At first, the salt stress affected the membrane transport (Foster and Miklavcic, 2015) via primary active proton pumps, secondary active antiporters and symporters, as well as passive ion channels. High salinity inhibits also plant growth via increasing osmotic pressure disrupting the plant ability to take up water and hence nutrients. Next, accumulation of salt ions (usually Na^+^) leads to concentrations that are toxic (Munns and Tester, 2008). The maintenance of a high cytosolic K^+^/Na^+^ ratio appears to be critical to plant salt tolerance (Shabala and Cuin, 2008). Exposure to high concentrations of Na^+^ can also disrupt the homeostasis of K^+^, which is essential for many physiological processes including excitability and organ movement.

### Strong but not mild salt stress decreases glu-induced series of APs

Besides ions, nitrogen is a major factor of plant growth (Debouba et al., 2006). The sensitivity to nitrogen sources in soil and capability of nitrogen uptake are basic features of growing plants. Glutamate is a major amino acid involved both in nitrogen metabolism and in nitrogen signaling (Forde and Lea, 2007; Mousavi et al., 2013). Previously, we showed that lithium modulated Glu-induced excitability and CN in sunflower seedlings (Stolarz et al., 2015). Thus, we expected that also the external concentration of K, Na, and Cl could modulate the Glu-induced series of APs. Here, we have shown for the first time the modulation of Glu-induced excitation by salinity. Seedlings growing in distilled water did not generate SAPs under 3-day observations (Table 1), but these plants were able to generate Glu-induced series of APs (Figure 2D, Table 2). Strong salt stress disturbed significantly the Glu-induced series of APs (Figure 2D, Table 2). Thus, it has been shown that only strong salt stress, but not osmotic stress, decreased Glu-induced series of APs. These results suggested that Glu-induced APs could be a part of the signaling pathway in nitrogen metabolism.

## Conclusions

Ions are important components of the cytoplasm and vacuole of living cells; therefore, their content in the environment as well as sensing and acquisition by plants is essential for survival. Potassium, chlorine, and sodium are crucial for plasma membrane potential maintenance and thus excitability of organisms, cell volume, growth, and movements. We have demonstrated the physiological impact of osmotic and salt stress on electrical signal induction and propagation as well as on CN and elongation of hypocotyls in whole *H. annuus* plants. Identification of SAPs accompanying varied CN vigor in seedlings growing in the hydroponic medium opens a new avenue for studying the relation between plant excitability and movements under different environment nutrient condition.

## Author contributions

MS designed and carried out the experiments, collected, and analyzed the results, and wrote the manuscript. HD helped in the analysis of the results and editing the manuscript.

### Conflict of interest statement

The authors declare that the research was conducted in the absence of any commercial or financial relationships that could be construed as a potential conflict of interest.
