# Supplementary material for: Osmotic and Salt Stresses Modulate Spontaneous and Glutamate-Induced Action Potentials and Distinguish between Growth and Circumnutation in Helianthus annuus Seedlings
Source: Front Plant Sci. 2017 Oct 18;8:1766. doi: 10.3389/fpls.2017.01766 (PMC5651625; doi:10.3389/fpls.2017.01766)
Supplement: Supplementary file 3 [file DataSheet1.PDF]

Supplementary Material

Osmotic and salt stresses modulate spontaneous and glutamate-induced action potentials and distinguish between growth and circumnutation in *Helianthus annuus* seedlings

Maria Stolarz*, Halina Dziubińska

* Corresponding author, Maria Stolarz
Department of Biophysics
Institute of Biology and Biochemistry
Maria Curie-Skłodowska University
Akademicka 19
20-033 Lublin, Poland
Tel.: (+48-81) 537 50 80
Fax: (+48-81) 537 59 01

Correspondence:
Maria Stolarz
e-mail: maria.stolarz@poczta.umcs.lublin.pl

Halina Dziubińska
Department of Biophysics
Institute of Biology and Biochemistry
Maria Curie-Skłodowska University
Akademicka 19
20-033 Lublin, Poland

Supplementary Figure S1. A scheme of the experimental arrangement for the recording of action potentials in *Helianthus annuus* (A) sunflower seedlings in a Faraday cage **(B)** computer (PC) with a multi-channel data acquisition system (das). The electrical measurements were carried out in a Faraday cage on 7- to 8-day-old seedlings or 3-week-old sunflowers. The changes in the electrical potential were measured with two (el.1, el.2) or four extracellular Ag/AgCl electrodes (a silver wire, 0.2 mm diameter, World Precision Instruments, Sarasota, FL, USA) inserted across the sunflower hypocotyl or stem and then interfaced with a multi-channel data acquisition system (das) composed of a differential amplifier (ME-4600 Meilhaus, Germany) and RealView software (Abacom, Germany). The reference electrode (Ag/AgCl, el. ref) was placed in the hydroponic medium or soil. (i) site of solution injection. The frequency of sample recording was 1 Hz. For registration of SAPs (spontaneous action potentials), seedlings grew in the Faraday cage in a hydroponic solution for three days. Glutamate (Glu) injection was applied between 8:00 a.m.-2:00 p.m. in 7-8-day-old seedlings growing in different nutrient solutions for three days.

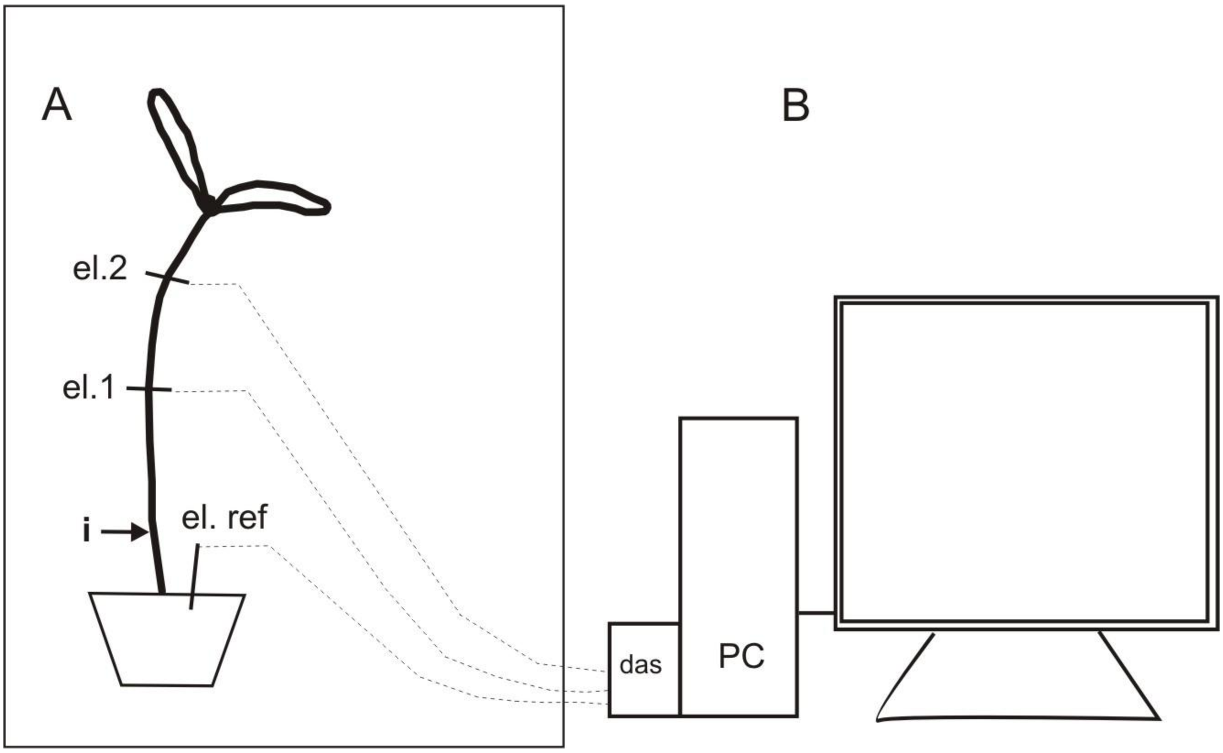

Supplementary Figure S2. Sunflower seedlings under osmotic and salt stress. (A) Seedlings in a hydroponic culture. **(B)** View of seedlings after measurement. Data details are presented in Figure 1.

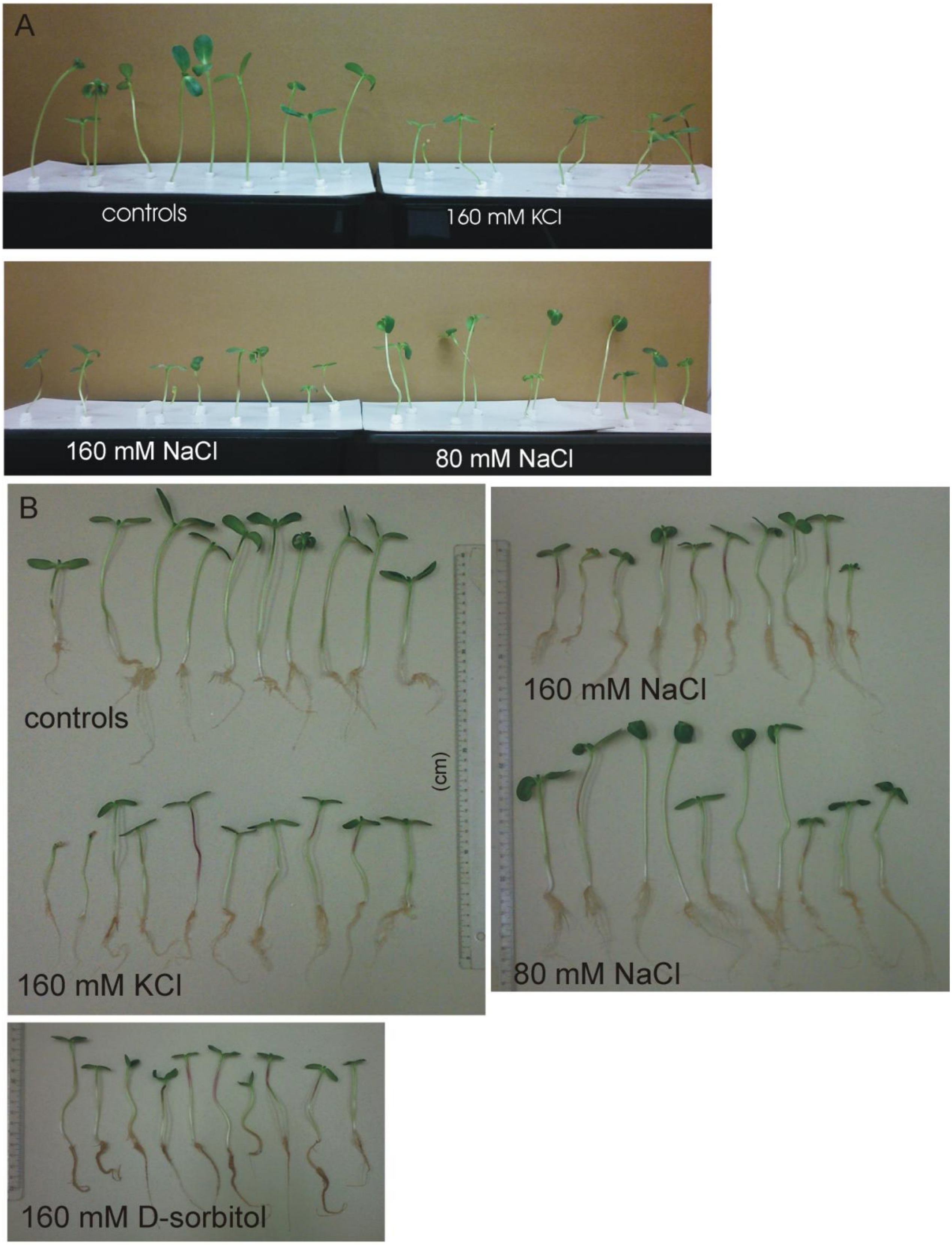

Supplementary Figure S3. Distilled water and sodium chloride-induced electrical potential changes in three-week old *Helianthus annuus*. (A) Example of recordings of action potential series after distilled water injection into the stem base. **(B)** Example of recordings of electrical potential changes after NaCl 500 mOsm (250 mM) injection. *Helianthus annuus* plants, electrode arrangement (1, 2, 3, 4, ref – reference electrode), and site of solution injection (i) are shown in Figure 3A. Data details are presented in Table 3.

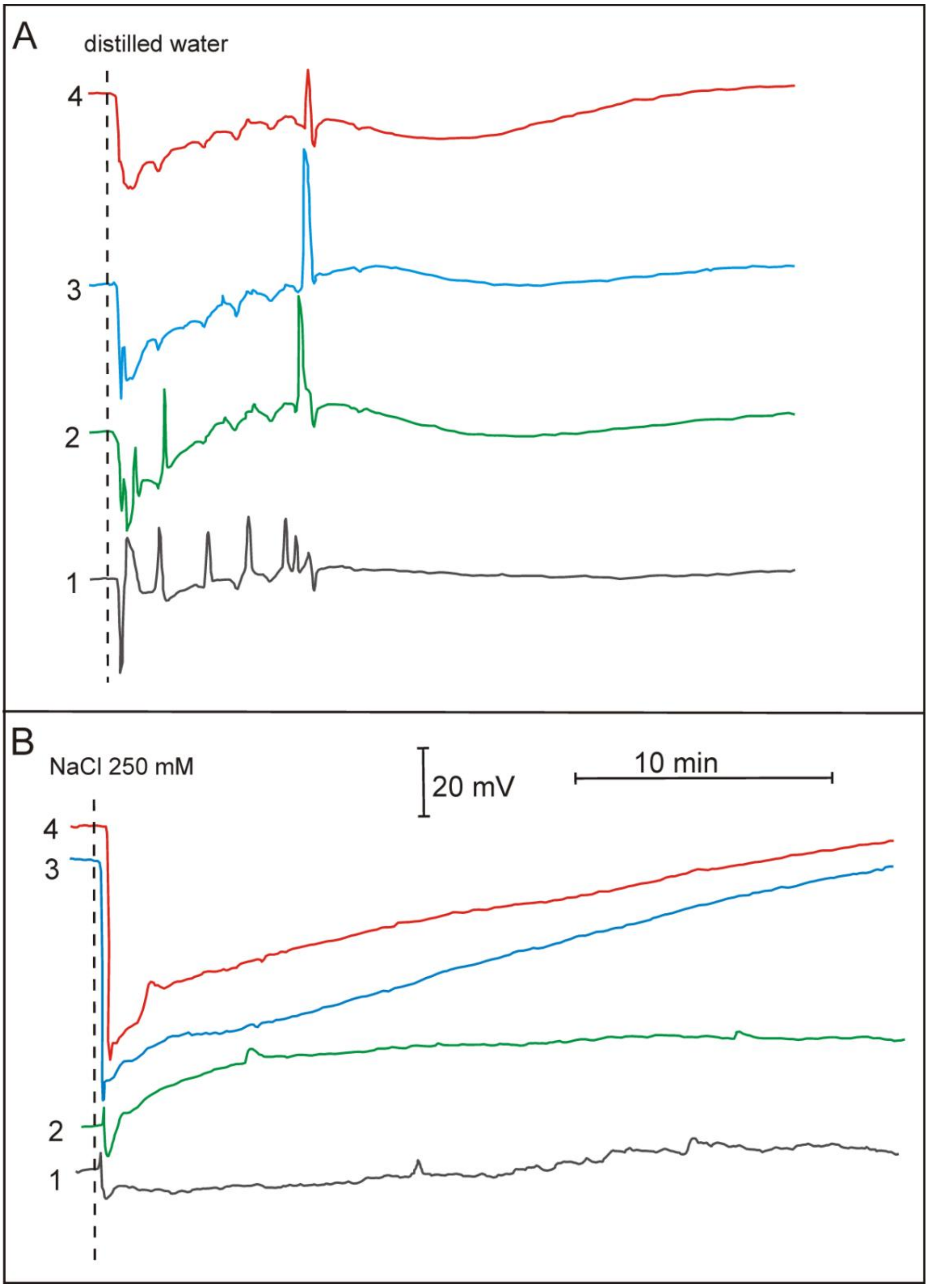

Supplementary Video S1. Circumnutation cycles in *Helianthus annuus* seedlings during 24 hours. In the geographic direction plane (top view), the single circumnutation cycle is determined by two subsequent maximum northward bends of the organ (Stolarz et al., 2014). On the video the maximum northward bend of the hypocotyl is marked by the yellow arrow.

Supplementary Video S2. Spontaneous action potentials in *Helianthus annuus* seedlings Example of original electrophysiological recording of spontaneous action potentials (SAP) during one day in a seedling growing in hydroponic nutrient containing 80 mM NaCl. *Helianthus annuus* seedlings and electrode arrangement (el. 1 red line, el. 2 green line, ref – reference electrode) are shown in Figure 2A. All SAPs propagated basipetally. SAPs appeared at approx. the following hours: 11:39 a.m., 1:38 p.m., 3:28 p.m., 5:45 p.m., 7:43 p.m., 8:16 p.m., 11:22 p.m., 3:01 a.m., 8:56 a.m., 9:23 a.m..
